# Inhibition of tetrodotoxin-resistant sodium current in dorsal root ganglia neurons mediated by D1/D5 dopamine receptors

**DOI:** 10.1186/1744-8069-9-60

**Published:** 2013-11-28

**Authors:** William Galbavy, Elham Safaie, Mario J Rebecchi, Michelino Puopolo

**Affiliations:** 1 Department of Anesthesiology, Stony Brook Medicine, Stony Brook, NY 11794, USA; 2 Department of Radiology, Stony Brook Medicine, Stony Brook, NY 11794, USA; 3 Department of Anesthesiology, Stony Brook Medicine, Health Sciences Center L4-072, Stony Brook, NY 11794, USA

**Keywords:** Dorsal root ganglia, D1/D5 dopamine receptors, Tetrodotoxin-resistant sodium channels

## Abstract

**Background:**

Dopaminergic fibers originating from area A11 of the hypothalamus project to different levels of the spinal cord and represent the major source of dopamine. In addition, tyrosine hydroxylase, the rate-limiting enzyme for the synthesis of catecholamines, is expressed in 8-10% of dorsal root ganglia (DRG) neurons, suggesting that dopamine may be released in the dorsal root ganglia. Dopamine has been shown to modulate calcium current in DRG neurons, but the effects of dopamine on sodium current and on the firing properties of small DRG neurons are poorly understood.

**Results:**

The effects of dopamine and dopamine receptor agonists were tested on the tetrodotoxin-resistant (TTX-R) sodium current recorded from acutely dissociated small (diameter ≤ 25 μm) DRG neurons. Dopamine (20 μM) and SKF 81297 (10 μM) caused inhibition of TTX-R sodium current in small DRG neurons by 23% and 37%, respectively. In contrast, quinpirole (20 μM) had no effects on the TTX-R sodium current. Inhibition by SKF 81297 of the TTX-R sodium current was not affected when the protein kinase A (PKA) activity was blocked with the PKA inhibitory peptide (6–22), but was greatly reduced when the protein kinase C (PKC) activity was blocked with the PKC inhibitory peptide (19–36), suggesting that activation of D1/D5 dopamine receptors is linked to PKC activity. Expression of D1and D5 dopamine receptors in small DRG neurons, but not D2 dopamine receptors, was confirmed by Western blotting and immunofluorescence analysis. In current clamp experiments, the number of action potentials elicited in small DRG neurons by current injection was reduced by ~ 30% by SKF 81297.

**Conclusions:**

We conclude that activation of D1/D5 dopamine receptors inhibits TTX-R sodium current in unmyelinated nociceptive neurons and dampens their intrinsic excitability by reducing the number of action potentials in response to stimulus. Increasing or decreasing levels of dopamine in the dorsal root ganglia may serve to adjust the sensitivity of nociceptors to noxious stimuli.

## Background

Small DRG neurons with unmyelinated axons signal nociceptive sensory information from the body surface and viscera to the dorsal spinal horn. Small DRG neurons are unusual in expressing both tetrodotoxin-sensitive (TTX-S) (Na_V_1.1, Na_V_1.2, Na_V_1.6, and Na_V_1.7) and TTX-R (Na_V_1.8 and Na_V_1.9) voltage-dependent sodium channels [1-9]. Na_V_1.8 and Na_V_1.9 channels are preferentially expressed in small DRG neurons and fulfill critical aspects of their physiology [10-14]. In addition to their contribution to different aspects of pain [15-21], Na_V_1.8 channels play a major role during the upstroke of the action potential in small DRG neurons [14,22,23], and thus represent an ideal target for neuromodulators to affect their intrinsic excitability.

Dopaminergic fibers from area A11 of the hypothalamus project to different levels of the spinal cord and represent the major source of dopamine [24-28]. A functional role of dopamine in the spinal cord is suggested by: *i*) expression of D1-D5 dopamine receptors [29-31]; *ii*) peripheral nocifensive action of dopamine [32-37]; *iii*) post-synaptic effects of dopamine in the dorsal spinal horn that can inhibit nociceptive transmission [38,39].

In addition to descending dopaminergic fibers from the hypothalamus, several subpopulations of DRG neurons, including those specialized in detecting low-threshold mechanosensory stimuli and those innervating pelvic organs, express tyrosine hydroxylase (TH), the rate-limiting enzyme for the synthesis of catecholamines, and thus most likely release dopamine as a modulatory transmitter [40-45]. Cultured DRG neurons release various peptides and ATP from their soma [46-48], suggesting that they may also release dopamine as well [49].

Dopamine has been shown to modulate calcium current in DRG neurons [50-52], but the effects of dopamine on sodium current and on the intrinsic excitability of small DRG neurons are poorly understood. Despite some reports showing inhibition of sodium current in chick sensory neurons [50], intracellular recordings from DRG neurons in isolated spinal ganglia have reported contrasting results showing depolarization, hyperpolarization or biphasic responses of the cell membrane upon application of dopamine [53-55].

Here, using an *in vitro* preparation of acutely dissociated DRG neurons, we show that dopamine inhibits the TTX-R sodium current in small DRG neurons and dampens their intrinsic excitability. Pharmacological studies indicate that the effect of dopamine is mediated by D1/D5 dopamine receptors.

## Results

### Dopamine effect on TTX-resistant sodium current

**Figure 1:**
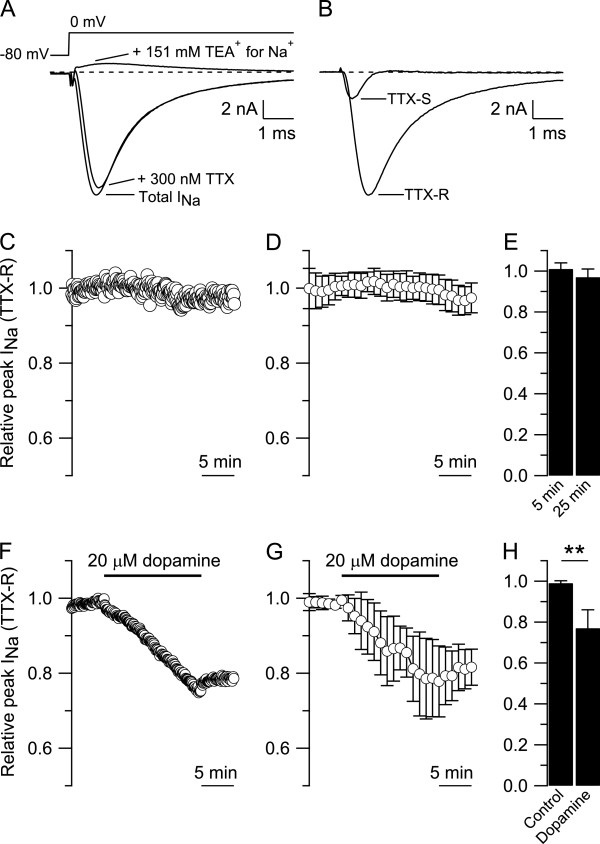
**Dopamine effect on TTX-R sodium current. A)** Isolation of TTX-R sodium current from a small DRG neuron (diameter ≤ 25 μm). Top: voltage clamp protocol. A single step of voltage from −80 to 0 mV, 30 msec duration, was delivered every 5 sec. Bottom: Total sodium current (Total I_Na_) recorded in Tyrode’s solution supplemented with 30 μM CdCl_2_ to block calcium current. Subsequent application of 300 nM tetrodotoxin blocked the TTX-S sodium current leaving only the TTX-R sodium current which was completely blocked when 151 mM NaCl was replaced by equimolar concentration of tetraethylammonium-Cl. **B)** TTX-S and TTX-R sodium currents isolated by subtraction from the cell in A. **C)** The TTX-R sodium current recorded in a small DRG neuron was normalized to the peak TTX-R sodium current recorded during the first 5 min in whole-cell and monitored for 25 min. **D)** Collected results showing the relative peak of TTX-R sodium current during 25 min. **E)** The relative peak of TTX-R sodium current was 1.01 ± 0.03 at 5 min and 0.97 ± 0.04 at 25 min (n = 11, paired t-Test, p = 0.104). **F)** In a small DRG neuron, 20 μM dopamine reduced the relative peak of TTX-R sodium current from 0.99 (after 5 min in control) to 0.75 (after 15 min in 20 μM dopamine). **G)** Collected results showing the effect of 20 μM dopamine on the relative peak of TTX-R sodium current. **H)** The relative peak of TTX-R sodium current was reduced from 0.99 ± 0.01 after 5 min in control to 0.77 ± 0.09 (n = 7, paired t-test, **p < 0.01) after 15 min in 20 μM dopamine.

Small DRG neurons express both TTX-S and TTX-R sodium channels. In order to isolate the TTX-R sodium current, TTX-S sodium current was blocked by 300 nM TTX in the presence of 30 μM Cd^2+^ to block calcium current. The inward current remaining in 300 nM TTX was completely blocked when 151 mM NaCl was replaced with equimolar tetraethylammonium-Cl (Figure 1A). Tetrodotoxin (300 nM) was only a weak inhibitor of the total sodium current, reducing the peak by 19.6 ± 14.0% (n = 32), similar to previous reports in small DRG neurons [9,23]. The TTX-R sodium current isolated with this protocol (Figure 1B) is consistent with sodium current carried through Na_V_1.8 channels previously reported in small DRG neurons [1-5,56]. At the holding potential of −80 mV, there was no obvious component of non-inactivating current from Na_V_1.9 channels. The TTX-R sodium current recorded with this protocol was quite stable over 20–25 min (Figure 1C). In collected results (Figure 1D,E), the TTX-R sodium current showed no significant decrease during 25 min, changing from 1.01 ± 0.03 (normalized peak) during the first 5 min to 0.97 ± 0.04 after 25 min (n = 11, paired t-test, p = 0.104). We next used this same protocol to test the effect of dopamine on TTX-R sodium current. After 5 min in control, the neuron was challenged with 20 μM dopamine (Figure 1F). Dopamine caused a clear inhibition of TTX-R sodium current. In collected results (Figure 1G,H), the TTX-R sodium current was reduced from 0.99 ± 0.01 to 0.77 ± 0.09 (n = 7, paired t-test, **p < 0.01) after 15 min in 20 μM dopamine.

### Effect of SKF 81297 on TTX-R sodium current

**Figure 2:**
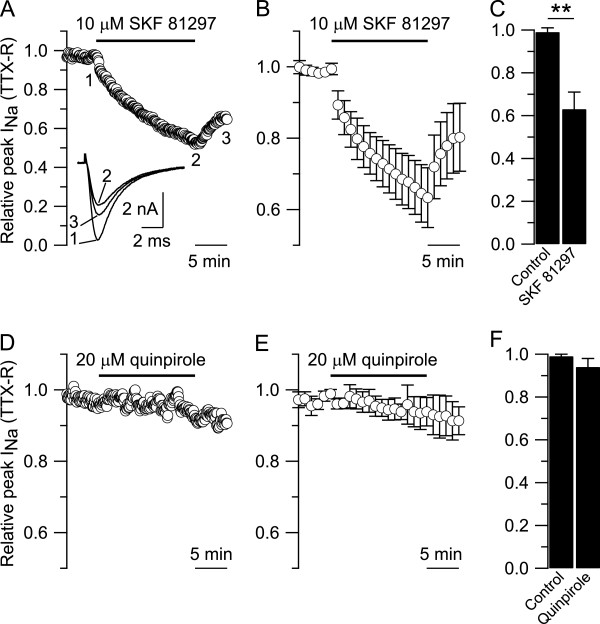
**Effect of D1/D5 and D2 dopamine receptor agonists on TTX-R sodium current. A)** In a small DRG neuron (diameter ≤ 25 μm), 10 μM SKF 81297 (D1/D5 dopamine receptors agonist) reduced the relative peak of TTX-R sodium current from 0.97 after 5 min in control to 0.51 after 15 min in SKF 81297. Inset: representative TTX-R sodium current traces recorded after 5 min in control (trace 1), after 15 min in 10 μM SKF 81297 (trace 2), and after 5 min washout (trace 3). **B)** Collected results showing the effect of 10 μM SKF 81297 on the relative peak of TTX-R sodium current. **C)** The relative peak of TTX-R sodium current was reduced from 0.99 ± 0.02 after 5 min in control to 0.63 ± 0.08 (n = 22, paired t-Test, **p < 0.01) after 15 min in 10 μM SKF 81297. **D)** In a small DRG neuron, 20 μM quinpirole (D2 dopamine receptor agonist) caused only a little change on the relative peak of TTX-R sodium current from 0.97 after 5 min in control to 0.94 after 15 min in 20 μM quinpirole. **E)** Collected results showing the effect of 20 μM quinpirole on the relative peak of TTX-R sodium current. **F)** The relative peak of TTX-R sodium current was reduced from 0.99 ± 0.01 after 5 min in control to 0.94 ± 0.04 (n = 5, paired t-test, p = 0.065) after 15 min in 20 μM quinpirole.

The next step was to identify which dopamine receptors mediate this inhibitory effect. The D1/D5 receptor agonist SKF 81297 produced inhibition very much like that of dopamine (Figure 2A). In collected results (Figure 2B,C), the TTX-R sodium current was reduced from 0.99 ± 0.02 to 0.63 ± 0.08 (n = 22, paired t-test, **p < 0.01) after 15 min in 10 μM SKF 81297. In contrast, the D2 receptor agonist quinpirole had no significant effect (Figure 2D); in collected results (Figure 2E,F), the TTX-R sodium current was reduced from 0.99 ± 0.01 to 0.94 ± 0.04 (n = 5, paired t-test, p = 0.065) after 15 min in 20 μM quinpirole. These results strongly suggest that the inhibitory effect of dopamine on TTX-R sodium current in small DRG neurons is mediated by activation of D1/D5 dopamine receptors.

**Figure 3:**
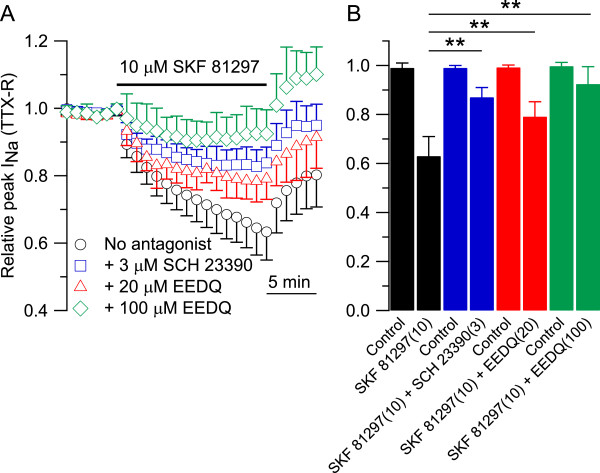
**The effect of SKF 81297 (D1/D5 dopamine receptors agonist) on TTX-R sodium current was antagonized by SCH 23390 and EEDQ. A)** Collected results showing the effect of 10 μM SKF 81297 on TTX-R sodium current applied either alone (black circles), or in combination with 3 μM SCH 23390 (blue squares), or 20 μM EEDQ (red triangles), or 100 μM EEDQ (green diamonds). **B)** The inhibitory effect of 10 μM SKF 81297 on the relative peak of TTX-R sodium current was reduced from 0.63 ± 0.08 (n = 22) when applied without the antagonist (black bars) to 0.83 ± 0.04 (n = 8, one-way ANOVA, followed by post-hoc Dunnett analysis **p < 0.01) when applied on DRG neurons incubated for 15 min with 3 μM SCH 23390 (blue bars); to 0.79 ± 0.06 (n = 6, one-way ANOVA, followed by post-hoc Dunnett analysis **p < 0.01) when applied on DRG neurons incubated for 30 min with 20 μM EEDQ (red bars); to 0.92 ± 0.07 (n = 9, one-way ANOVA, followed by post-hoc Dunnett analysis, **p < 0.01) when applied on DRG neurons incubated for 30 min with 100 μM EEDQ (green bars).

To test this further, we used SCH 23390, a selective antagonist at D1/D5 dopamine receptors [57,58]. As expected, the effect of SKF 81297 (Figure 3A, black circles) was antagonized by SCH 23390 (Figure 3A, blue squares). Similarly, the effect of SKF 81297 was also reduced by the alkylating agent ethoxycarbonyl-2-ethoxy-1,2-dihydroquinoline (EEDQ), a broad, irreversible, dopamine receptor antagonist [Figure 3A, 20 μM EEDQ (red triangles) and 100 μM EEDQ (green diamonds)]. In collected results (Figure 3B), the inhibitory effect of 10 μM SKF 81297 on TTX-R sodium current was reduced from 0.63 ± 0.08 (n = 22) when applied without the antagonist (black bars) to 0.83 ± 0.04 (n = 8, one-way ANOVA, followed by Dunnett post-hoc analysis **p < 0.01) when applied in combination with 3 μM SCH 23390 (blue bars); to 0.79 ± 0.06 (n = 6, one-way ANOVA, followed by Dunnett post-hoc analysis, **p < 0.01) when applied in combination with 20 μM EEDQ (red bars); to 0.92 ± 0.07 (n = 9, one-way ANOVA, followed by Dunnett post-hoc analysis, **p < 0.01) when applied in combination with 100 μM EEDQ (green bars).

### Intracellular pathways downstream to D1/D5 dopamine receptors

**Figure 4:**
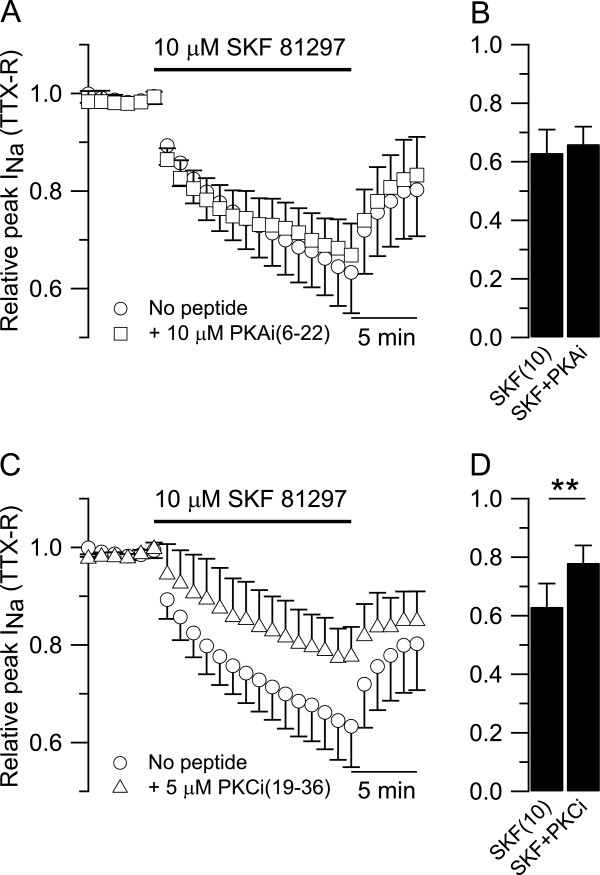
**Intracelluar pathway downstream to D1/D5 dopamine receptors activation. A)** Collected results showing the effect of 10 μM SKF 81297 on TTX-R sodium current applied either alone (circles) or in combination with 10 μM PKAi(6–22) (squares) included in the patch pipette to block the PKA activity. **B)** The inhibitory effect of 10 μM SKF 81297 on the relative peak of TTX-R sodium current was slightly reduced from 0.63 ± 0.08 (n = 22) when applied alone to 0.66 ± 0.06 (n = 11, t-test, two populations, p = 0.229) when applied in combination with 10 μM PKAi(6–22) included in the patch pipette. **C)** Collected results showing the effect of 10 μM SKF 81297 on TTX-R sodium current applied either alone (circles) or in combination with 5 μM PKCi(19–36) (triangles) included in the patch pipette to block the PKC activity. **D)** The inhibitory effect of 10 μM SKF 81297 on the relative peak of TTX-R sodium current was significantly reduced from 0.63 ± 0.08 (n = 22) when applied alone to 0.78 ± 0.06 (n = 11, t-test, two populations, **p < 0.01) when applied in combination with 5 μM of PKCi(19–36) included in the patch pipette.

Previous work has described the ability of PKA and PKC to modulate both TTX-S sodium current in central neurons [57-59] and TTX-R sodium current in peripheral sensory neurons [60-65]. When PKA activity was blocked by including in the patch pipette 10 μM of PKA inhibitory fragment (6–22) [PKAi(6–22)], the inhibitory effect of 10 μM SKF 81297 on TTX-R sodium current in small DRG neurons was unaffected. In collected results (Figure 4A,B), the TTX-R sodium current was reduced from 0.99 ± 0.01 to 0.63 ± 0.08 (n = 22) after 15 min in 10 μM SKF 81297 (Figure 4A, circles) and from 0.99 ± 0.01 to 0.66 ± 0.06 (n = 11, t-test, two populations, p = 0.229) after 15 min in 10 μM SKF 81297 with 10 μM PKAi(6–22) included in the patch pipette (Figure 4A, squares).

In contrast, when PKC activity was blocked by including in the patch pipette 5 μM of PKC inhibitory fragment (19–36) [PKCi(19–36)], the inhibitory effect of 10 μM SKF 81297 on the TTX-R sodium current was substantially affected. In collected results (Figure 4C,D), the TTX-R sodium current was reduced from 0.99 ± 0.01 to 0.63 ± 0.08 (n = 22) after 15 min in 10 μM SKF 81297 (Figure 4C, circles) and from 0.99 ± 0.01 to 0.78 ± 0.06 (n = 11, t-test, two populations, **p < 0.01) after 15 min in 10 μM SKF 81297 with 5 μM of PKCi(19–36) included in the patch pipette (Figure 4C, triangles). Taken together, these results suggest that activation of D1/D5 dopamine receptors in small DRG neurons is coupled to PKC activity.

### Effect of SKF 81297 on steady-state activation and inactivation curves of TTX-R sodium channels

**Figure 5:**
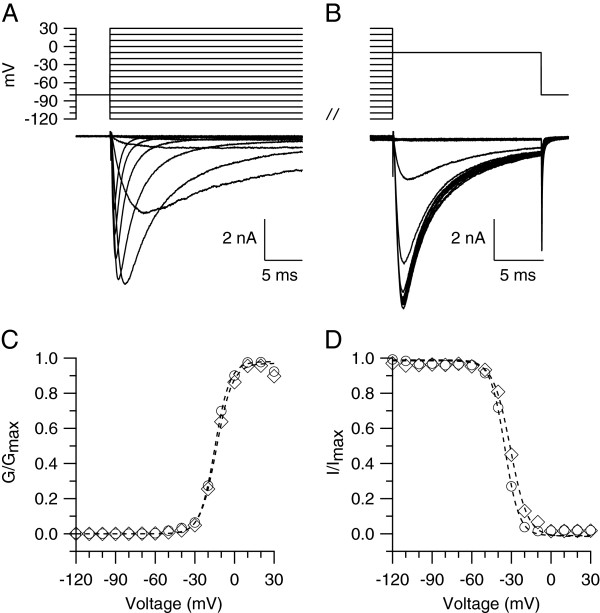
**Effect of SKF 81297 on the steady-state activation and inactivation curves of TTX-R sodium channels. A)** Voltage-dependence activation of TTX-R sodium channels. TTX-R voltage-gated sodium currents were elicited by 500 msec steps from a holding potential of – 80 mV to voltages between −120 and + 30 mV in 10 mV increments. **B)** Voltage-dependence of inactivation of TTX-R sodium channels. TTX-R voltage-gated sodium currents were elicited with a test pulse to −10 mV preceded by 500 msec prepulses from −120 to + 30 mV in 10 mV increments. **C)** Collected results showing the effect of 10 μM SKF 81297 on the steady-state activation curve of TTX-R sodium channels in small DRG neurons (diameter ≤ 25 μm). The steady-state activation curve had a midpoint for activation of −15.1 ± 4.2 mV and a slope of 5.0 ± 1.8 mV (n = 17) in control (circles) versus −12.7 ± 7.4 mV (t-test, two populations, p = 0.270) and 4.9 ± 2.1 mV (n = 15) in neurons incubated for 15 min in 10 μM SKF 81297 (diamonds). Dotted lines are best fits to the Boltzmann function. Error bars were omitted for clarity. **D)** Collected results showing the effect of 10 μM SKF 81297 on the steady-state inactivation curve of TTX-R sodium channels (same neurons as in **C**). The steady-state inactivation curve had a midpoint for inactivation of −34.7 ± 3.2 mV and a slope of 4.9 ± 1.1 mV (n = 17) in control (circles) versus −31.3 ± 4.2 mV (t-test, two populations, *p < 0.05) and 5.2 ± 1.9 mV (n = 15) in 10 μM SKF 81297 (diamonds). Dotted lines are best fits to the Boltzmann function. Error bars were omitted for clarity.

We next tested whether D1/D5 dopamine receptors inhibition of TTX-R sodium channels is mediated by changes in the voltage-dependence of activation or inactivation of the channels. The steady-state activation curve had a midpoint of −15.1 ± 4.2 mV and a slope of 5.0 ± 1.8 mV (n = 17) in control (Figure 5C, circles) versus −12.7 ± 7.4 mV (t-test, two populations, p = 0.270) and 4.9 ± 2.1 mV (n = 15) in 10 μM SKF 81297 (Figure 5C, diamonds). The steady-state inactivation curve had a midpoint of −34.7 ± 3.2 mV and a slope of 4.9 ± 1.1 mV (n = 17) in control (Figure 5D, circles) versus −31.3 ± 4.2 mV (t-test, two populations, *p < 0.05) and 5.2 ± 1.9 mV (n = 15) in 10 μM SKF 81297 (Figure 5D, diamonds). The changes induced by SKF 81297 on the steady-state activation and inactivation curves of TTX-R sodium channels are similar to previously reported effects of D1/D5 dopamine receptors activation on TTX-S sodium channels both in central neurons [57-59] and in retinal ganglion cells [66].

### Effect of SKF 81297 on firing

**Figure 6:**
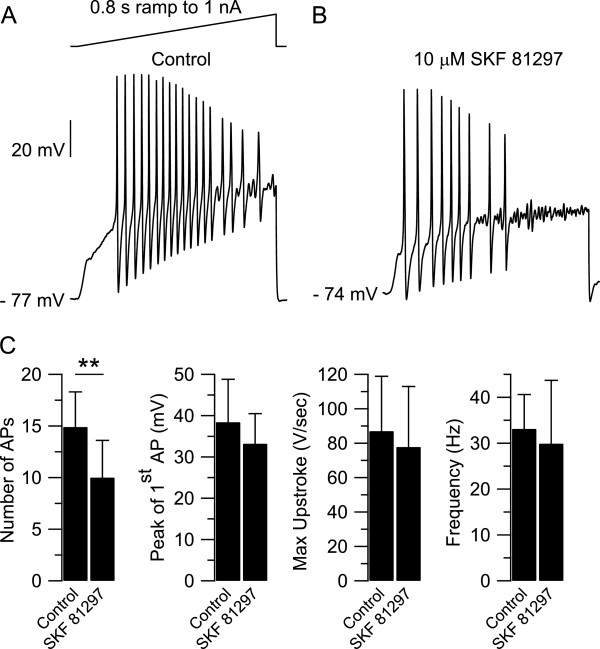
**Effect of SKF 81297 on the firing properties of small DRG neurons. A)** Top: current clamp protocol: 0.8 sec ramp of current to 1 nA was injected to elicit action potentials. Bottom: action potentials elicited in a small DRG neuron (diameter ≤ 25 μm) in control. **B)** Action potentials elicited in a different small DRG neuron (diameter ≤ 25 μm) incubated for 15 min in 10 μM SKF 81297. **C)** In collected results, the total number of action potentials during the first current injection decreased from 14.9 ± 3.4 (n = 15) in control to 10.0 ± 3.6 (n = 12) in 10 μM SKF 81297 (t-test, two populations **p < 0.01); the peak of the first action potential decreased from 38.4 ± 10.4 mV (n = 15) in control to 33.2 ± 7.3 mV (n = 12) in 10 μM SKF 81297 (t-test, two populations, p = 0.137); the maximum upstroke velocity of the first action potential decreased from 87.0 ± 31.9 V/sec (n = 15) in control to 77.8 ± 35.2 V/sec (n = 12) in 10 μM SKF 81297 (t-test, two populations, p = 0.484); the firing frequency decreased from 33.1 ± 7.5 Hz (n = 15) in control to 29.9 ± 13.8 Hz (n = 12) in 10 μM SKF 81297 (t-test, two populations, p = 0.459).

The TTX-R sodium current plays a major role during the upstroke of the action potential in small DRG neurons [14,22,23], and thus may represent an ideal target for neuromodulators to induce changes in the intrinsic excitability of nociceptors. In order to test this hypothesis, the effect of SKF 81297 was assessed in current clamp in which trains of action potentials were elicited in small DRG neurons by current injection [56]. During the initial control experiments in current clamp, we observed a progressive reduction in the number of action potentials elicited with repeated current injections. The number of action potentials decreased from 15.3 ± 3.8 (n = 6) during the first ramp to 9.8 ± 2.5 (n = 6) during the fifth ramp, even with 30 sec interval between current injections, possibly reflecting accumulation of slow inactivation of sodium channels during sustained depolarization [56]. For this reason, we decided to limit the analysis to action potentials elicited during the first current injection by making a comparison between small DRG neurons in control to those incubated with 10 μM SKF 81297 for 15 min (Figure 6A,B). The resting potential of small DRG neurons in control (−73.6 ± 4.9 mV, n = 15) was very similar to the resting potential of small DRG neurons incubated for 15 min in 10 μM SKF 81297 (−73.7 ± 5.0 mV, n = 12, t-test, two populations, p = 0.978). The total number of action potentials during the first current injection decreased from 14.9 ± 3.4 (n = 15) in control to 10.0 ± 3.6 (n = 12, t-test, two populations **p < 0.01) in 10 μM SKF 81297. Although not statistically significant, there appeared to be also a decrease in the peak of the first action potential from 38.4 ± 10.4 mV (n = 15) in control to 33.2 ± 7.3 mV (n = 12, t-test, two populations, p = 0.137) in 10 μM SKF 81297; a decrease in the maximum upstroke velocity of the first action potential from 87.0 ± 31.9 V/sec (n = 15) in control to 77.8 ± 35.2 V/sec (n = 12, t-test, two populations, p = 0.484) in 10 μM SKF 81297; a decrease in the firing frequency from 33.1 ± 7.5 Hz (n = 15) in control to 29.9 ± 13.8 Hz (n = 12, t-test, two populations, p = 0.459) in 10 μM SKF 81297 (Figure 6C). Taken together, these results suggest that activation of D1/D5 dopamine receptors induces substantial changes in the intrinsic excitability of small DRG neurons and reduces their ability to sustain volleys of action potentials in response to current injection.

### Expression of dopamine receptors in small DRG neurons

**Figure 7:**
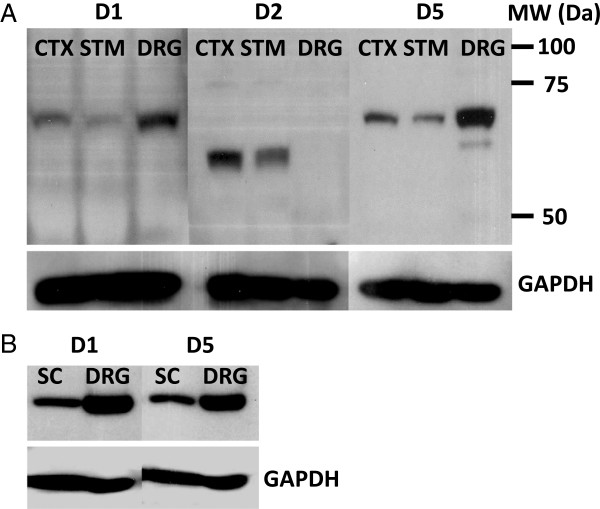
**Western blotting of dopamine receptors.** Immunoblots for D1, D2, and D5 dopamine receptors were obtained for frontal cortex (CTX), striatum (STM), spinal cord (SC) and dorsal root ganglia (DRG) proteins. All wells were loaded with the same concentration of total protein with GAPDH serving as an internal control. **A)** Membranes were probed with antibodies specific for D1, D2, or D5 dopamine receptors. D1 and D5 were identified in CTX, STM, and DRG as single bands with apparent MW’s of 68 kDa. D2 dopamine receptor protein was identified in CTX and STM as a single band with an apparent MW of 62 kDa. Corresponding sections from each blot were probed with antibody against GAPDH (shown beneath the membranes in **A**). **B)** Western blotting of D1 and D5 dopamine receptors were repeated comparing equal concentrations of SC and DRG protein. Corresponding sections from each blot were probed with antibody against GAPDH (shown beneath the membranes in **B**).

**Figure 8:**
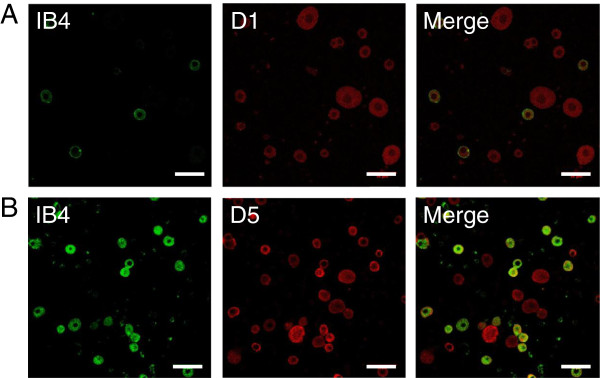
**Expression of dopamine receptors in acutely isolated DRG neurons.** Acutely dissociated DRG neurons were fixed in 4% formaldehyde, permeabilized, blocked and then probed with lectin FITC-labeled IB4 (green channel) or D1 (red channel, **A**) or D5 dopamine receptors antibodies (red channel, **B**) and secondary antibodies against mouse IgG labeled with Alexa 594. Merging the images indicated some co-localization of IB4 and either D1 or D5 dopamine receptors (yellow). Scale bars shown are 50 μm.

**Figure 9:**
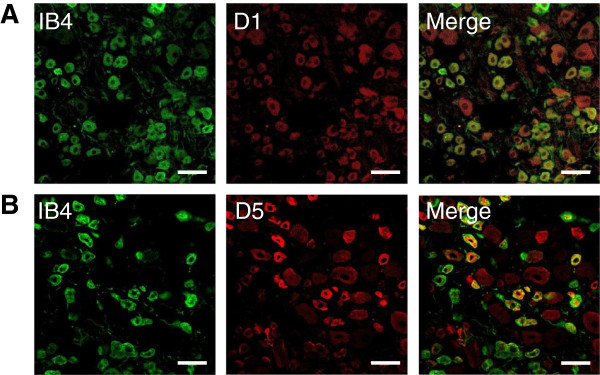
**Expression of dopamine receptors in intact dorsal root ganglia.** Frozen sections, 20 μm thick, were prepared from lumbar DRG taken from P14 to P28 Sprague Dawley rats perfused with formaldehyde. Sections of dorsal root ganglia were stained with FITC-labeled lectin IB4 (green channel) and monoclonal antibodies to either D1 (red channel, **A**) or D5 dopamine receptors antibodies (red channel, **B**), and secondary antibodies against mouse IgG labeled with Alexa 594. Merging the images indicated some co-localization of IB4 and either D1 or D5 dopamine receptors (yellow). Scale bars are 50 μm.

Our electrophysiology data strongly suggest that the effect of dopamine on TTX-R sodium current in small DRG neurons is mediated by activation of D1/D5 dopamine receptors, without any significant contribution of D2 dopamine receptors. To further test this hypothesis, we characterized the expression of D1, D2, and D5 dopamine receptors in DRG neurons. Relative tissue expression levels of D1, D2, and D5 dopamine receptor subtypes were evaluated by Western blotting. Surprisingly, levels of D1 and D5 dopamine receptors were substantially higher in dorsal root ganglia compared to striatum, cortex, or lumbar spinal cord. In contrast, D2 dopamine receptors were undetected in dorsal root ganglia but, highly enriched in striatum and cortex (Figure 7). Immunofluorescence imaging of dissociated DRG neurons showed that nearly all small DRG neurons (diameter ≤ 30 μm), both isolectin B4 positive [IB4(+)] and IB4(−) neurons, expressed D1 and D5 dopamine receptors. In contrast, most DRG neurons with diameter ≥ 30 μm were only faintly stained (Figure 8). While some plasma membrane staining was observed in small DRG neurons, most of the immunofluorescence signal was intracellular, apparently vesicular. The immunofluorescence results from fixed dissociated primary cultures were confirmed in fixed dorsal root ganglia sections (Figure 9). D1 and D5 dopamine receptors were primarily expressed in smaller DRG neurons. The intensity varied among the smaller neurons and no clear pattern of IB4(+) or IB4(−) association emerged. Although the immunofluorescence signals for D1 and D5 dopamine receptors in the larger IB4(−) neurons were above background, the overall intensities were much lower than those in smaller diameter neurons.

## Discussion

The data presented here show a strong inhibition of TTX-R sodium current in DRG neurons by activation of D1/D5 dopamine receptors, accompanied by decrease in their intrinsic excitability. Only small DRG neurons (diameter of 24.8 ± 2.6 μm and cell capacitance of 19.5 ± 4.4 pF, n = 111) sensitive to capsaicin were included in the study, and among those about 88% were also IB4(+). All these features suggest that the dopamine-sensitive neurons correspond to small nociceptive neurons [9,67-70].

Western blot and immunofluorescence analysis confirmed the expression of D1 and D5 dopamine receptors in dorsal root ganglia, but no evidence was found for the expression of D2 dopamine receptors. These results were consistent with those obtained with electrophysiology in which the D2 agonist quinpirole had little effect. Using polymerase chain reaction (PCR) and DNA sequencing, transcripts of D1-D5 dopamine receptors, including D2 dopamine receptors, have been reported in dorsal root ganglia [30,31]. A possible explanation for this discrepancy could be that, even though the transcript for D2 dopamine receptors is detectable with PCR, the protein level could be too low to be detected by Western blotting or immunofluorescence microscopy. Alternatively, it is possible that once translated into protein, D2 dopamine receptors are rapidly transported from the cell body to the terminals, and thus are missed with our analysis.

In most DRG neurons, the inhibitory effects of dopamine or SKF 81297 on TTX-R sodium current required many minutes to develop fully. In most of the neurons tested, the inhibitory effect of the drug did not reach a steady-state even after 15 min. This is slower than the inhibitory effect of dopamine on TTX-S sodium current in hippocampal or prefrontal cortex neurons [58,59], but similar to that in retinal ganglion cells [66]. The relative slow development of inhibition is consistent with mediation by a second messenger pathway involving PKC.

Activation of PKA or PKC has been reported to modulate Na_V_1.8 channels in DRG neurons with increases in current amplitude and changes in the kinetic properties [60-62]. Our results (Figure 4A) suggest that PKA activity is not required for modulation of TTX-R sodium current by D1/D5 dopamine receptor activation, in contrast with results in central neurons where activation of D1/D5 dopamine receptors inhibits the TTX-S sodium current through activation of PKA [57-59].

The reduced effect of SKF 81297 on TTX-R sodium current when blocking PKC (Figure 4C) strongly suggests that PKC activity is required for modulation of TTX-R sodium current upon activation of D1/D5 dopamine receptors. Activation [61,63,64] or inhibition [61] of PKC has been reported to up-regulate or down-regulate, respectively, the TTX-R sodium current in small DRG neurons. Even though multiple mechanisms/pathways are required to explain the full actions of PKC on TTX-R sodium current, the reduced effect of SKF 81297 on the TTX-R sodium current observed in our experiments is more consistent with inhibition of PKC activity upon activation of D1/D5 dopamine receptors. Additional experiments are needed to better characterize the intracellular pathway downstream to D1/D5 dopamine receptor activation in small DRG neurons.

The stimulation of D1/D5 dopamine receptors reduced the excitability of small DRG neurons, as manifested in reduction of action potential firing, and a decrease in the peak and the maximum upstroke velocity of action potentials. These effects are consistent with inhibition of TTX-R (Na_V_1.8) current, the main current responsible for the upstroke of the action potential in DRG neurons [14,22,23]. It is also likely, however, that D1/D5 dopamine receptor activation can modulate other ion channels critical to action potential generation in DRG neurons. Whether this could account for the changes observed here is unknown and will require further experiments.

Many observations support the idea that dopamine may be released locally in the dorsal root ganglia: *i*) TH, the rate-limiting enzyme for the synthesis of catecholamines, is expressed in about 8-10% of DRG neurons [40,41,43,44]; *ii*) dopamine and its metabolites dihydroxyphenylacetic acid (DOPAC) and homovanillic acid (HVA) have been detected in DRG neurons and in dorsal spinal nerve roots, but not in satellite and Schwann cells [45,71,72]; *iii*) when DRG neurons are loaded with dopamine, dopamine release could be evoked by high potassium stimulation and detected by amperometric means, suggesting that DRG neurons may carry the necessary release machinery for dopamine [49]. A question however remains why previous work has failed to detect other enzymes, namely the aromatic acid decarboxylase (AADC) and dopamine β-hydroxylase (DBH), involved in the synthesis of catecholamines [40,44,73,74], even though in some cases AADC and DBH have been detected in TH(−) DRG neurons [44]. A possible explanation for this discrepancy could be that the protein levels of AADC and DBH are too low to be detected by immunocytochemistry. Alternatively, it is possible that TH(+) DRG neurons may synthesize L-DOPA, which after release is converted to dopamine by AADC localized in other cell types.

In other sensory systems, including the retina and the olfactory bulb, spontaneously active dopaminergic cells provide a tonic release of dopamine [75-78], which plays an important function in sensory adaptation. In the retina, dopamine sets the gain of the retinal networks for vision in bright light [79]. Similarly, in the olfactory bulb, dopamine plays an important function in setting the gain for odor discrimination [80-84]. TH(+) DRG neurons form a selective class of unmyelinated Low-Threshold mechanoreceptors (C-LTMRs) innervating hair follicles in the skin [41] and associated with light touch and injury-induced mechanical hypersensitivity [85,86]. TH(+) C-LTMRs are not spontaneously active, but in response to light mechanical force they respond with a train of action potentials [41] that can trigger dopamine release. Light touch able to induce stretch of the skin or deflection of hair follicles able to activate C-LTMRs is a very frequent experience during the daily activity, supporting the possibility that TH(+) C-LTMRs may release dopamine in the dorsal root ganglia very frequently. Thus, increasing or decreasing levels of dopamine in the dorsal root ganglia may serve to adjust the sensitivity of nociceptors to noxious stimuli, with increasing levels of dopamine raising the threshold for pain and reduced levels increasing the sensitivity to noxious stimuli. Future behavioral experiments using animal models of pain are needed to test this hypothesis.

## Conclusions

In summary, our results demonstrate that DRG neurons express D1 and D5 dopamine receptors, but not D2 dopamine receptors. Agonists of D1/D5 dopamine receptors cause robust inhibition of TTX-R sodium current in small DRG neurons and decrease their ability to fire action potentials in response to stimulus. A functional implication of the inhibitory effect of dopamine could be that, by modulating the TTX-R sodium current and the intrinsic excitability of DRG neurons, increasing or decreasing levels of dopamine may adjust the sensitivity of nociceptor sensory neurons to incoming sensory stimuli.

## Materials and methods

### Preparation of isolated Dorsal Root Ganglia neurons

Isolated DRG neurons were prepared from Sprague Dawley rats, postnatal day 14–28. Animal were anesthetized with isoflurane and decapitated. Both thoracic and lumbar segments of the spinal cord were removed and placed in a cold Ca^2+^, Mg^2+^-free Hank’s solution containing (in mM) 137 NaCl, 5.3 KCl, 0.33 Na_2_HPO_4_, 0.44 KH_2_PO_4_, 5 HEPES, 5.5 Glucose, pH = 7.4 with NaOH. The bone surrounding the spinal cord was removed and dorsal root ganglia were exposed and pulled out. After removing the roots, ganglia were chopped in half and incubated for 20 min at 34°C in Ca^2+^, Mg^2+^-free Hank’s solution containing 20 U/ml Papain (Worthington Biochemical, Lakewood, NJ) and 5 mM D,L-cysteine. Ganglia were washed and incubated for 20 min at 34°C in Ca^2+^, Mg^2+^-free Hank’s solution containing 3 mg/ml collagenase (Type I, Sigma-Aldrich, St. Louis, MO) and 4 mg/ml Dispase II (Boehringer Mannheim, Indianapolis, IN). Ganglia were then placed in Leibovitz’s L-15 medium (Invitrogen, San Diego, CA) supplemented with 10% fetal calf serum, 5 mM HEPES, and 50 ng/ml NGF (Invitrogen, San Diego, CA). Individual cells were dispersed by mechanical trituration using fire-polished Pasteur pipettes with decreasing bore size and plated on glass coverslip treated with 30–50 μg/ml poly-D-lysine. Cells were incubated in the supplemented L-15 solution at 34°C (in 5% CO_2_) for 2 hours, then stored at room temperature in Neurobasal medium (Gibco) and used over the next 4–6 hours. This protocol yields spherical cell bodies without neurites, which enhances voltage control of sodium current. The cells can be lifted from the cover slip after establishing the whole-cell configuration in order to facilitate rapid solution changes using flow pipes.

### Western blotting

Immunoblots were performed for D1, D2 and D5 dopamine receptor proteins in dorsal root ganglia, frontal cortex, striatum and spinal cord of P14 to P28 Sprague Dawley rats. Animals were euthanized and perfused with heparinized saline. The dissected nervous tissue samples were immediately frozen on dry ice and stored at −80°C. Proteins were extracted from the frozen samples with ice cold lysis buffer prepared with complete EDTA-free protease inhibitor cocktail tablets (Roche Pharmaceuticals), 20 mM Tris, 150 mM NaCl, 2.5 mM Na_4_P_2_O_7_, 1% Nonidet P40, 0.1% SDS and 1 mM of EDTA, NaF, PMSF, Na_3_VO_4_, and dithiothrietol. Three 2.3 mm diameter Zircona/Silica beads (Biospec) were added to the samples which were then homogenized in a Biospec Mini Bead Beater for one min. Samples were centrifuged at 4°C at 13,000 × g for 15 min and the supernatant fluids were saved. Total protein concentrations were determined using Bio Rad Protein Assay Dye Reagent Concentrate. After the addition of concentrated Laemmli buffer and heating to 80°C, equal amounts of total protein were subjected to SDS PAGE in a 10% polyacrylamide gel and transferred to PVDF membrane in a Semi-Dry Blot apparatus (BioRad). Membranes were blocked overnight with 5% non-fat milk in TBS at 4°C and probed with D1 (Millipore MAB5290), D2 (Millipore AB5084P) or D5 (Millipore MAB5292) dopamine receptor antibodies at 1:500 with 5% non-fat milk in TBS for 3 h. Internal control GAPDH antibody (Sigma G8795) was incubated at 1:5000 with 5% non-fat milk in TBS for 1 h. After primary antibody incubation, membranes were washed 3 times with 0.05% Tween-20 in Tris-buffered saline (TBS-T) for 10 min each wash. Secondary antibodies linked to HRP were diluted 1:8000 (Santa Cruz, L0312) and 1:5000 (Santa Cruz, L1911) with 5% non-fat milk in TBS and incubated with the washed membranes for 2 h at room temperature. Membranes were then washed 3 times with TBS-T as described above and then antibody binding was detected with ECL plus reagent (GE).

### Immunocytochemistry

Indirect immunofluorescence was used to assess expression of the receptor subtypes in dissociated DRG neurons in primary culture. DRG neurons were fixed with 4% formaldehyde in Hank’s Buffered Saline Solution (HBSS) for 20 min, washed 3 times in PBS, 10 min each, and then permeabilized with 0.2% Nonidet P40 in TBS for 20 minutes. After 3 more washes with TBS, 10 min each, cover slips were blocked for 1 h with blocking solution (10% Goat Serum, 1% BSA in TBS) and then probed with primary dopamine receptor antibody overnight in blocking solution (1:500), followed by secondary antibody (1:1000 Invitrogen Alexa Fluor 594 A11032) with or without FITC-labeled lectin IB4 for 1.5 h at room temperature. Following 3 washes with TBS, the coverslips were rinsed with deionized water and then mounted (Prolong Gold Antifade mounting fluid) on Superfrost Plus microscope slides (Fisher) and imaged with a confocal microscope (Olympus Fluoview1000).

For fixed tissue specimens, P14 to P28 Sprague Dawley rats were euthanized and perfused with heparinized saline, followed by 4% formaldehyde in PBS. Dissected specimens were then post fixed for 30 min in 4% formaldehyde in PBS before being transferred to a solution of 30% sucrose in PBS for overnight incubation at 4°C. Fixed tissues were embedded in optimum cutting temperature compound (OCT), frozen on dry ice and then sliced into 20 μm thick sections with a cryostat (Leica) and collected onto Superfrost Plus microscope slides. As soon as dry, the sections were stored up to several days at −20°C. Before the addition of antibody, the sections were permeabilized and blocked with 10% goat serum in TBS with 0.1% Nonidet P40 for 3 h at room temperature. Specimens were then probed with primary antibody overnight in 10% goat serum in TBS at 4°C. They were then washed 3 times in TBS-T and then incubated with fluorescently labeled secondary antibody (1:1000 Invitrogen Alexa Fluor 594 A11032) diluted with 10% goat serum and 1% rat serum in TBS with or without FITC-labeled IB4 for 2 h at room temperature. The wash steps were repeated. The slides were rinsed with deionized water and thoroughly drained. A drop of mounting fluid was placed on each section and coverslips were mounted. All slides were imaged on a confocal microscope (Olympus Fluoview 1000). Images were obtained with a 40× oil objective and 1.4 NA lens. All images were exported as TIFF files to Image J, which was used to process the final images.

### Cell classification

Small DRG neurons (cell diameter ≤ 25 μm) were chosen for recording. At the end of each experiment, the recorded DRG neuron was tested for the expression of TRPV1 channels (by testing the sensitivity to 1 μM capsaicin) and classified as nociceptor or non-nociceptor [87]. As nociceptors are neurochemically and functionally distinct [69,70,88,89], a further classification was made by testing the ability of TRPV1-positive DRG neurons to bind the isolectin B4 (IB4) FITC conjugate and classified as peptidergic [IB4(−)] or non-peptidergic [IB4(+)]. Overall about 75-80% of small DRG neurons tested were sensitive to capsaicin and only those were included in this study. Of the neurons sensitive to capsaicin, about 88% were also IB4(+). Small DRG neurons with these properties are considered to be nociceptive neurons [67-70].

### Electrophysiology

Whole-cell recordings were made with a Multiclamp 700B amplifier (Molecular Devices, Sunnyvale, CA). Patch pipettes were pulled from borosilicate glass (100 μl microcapillaries, VWR, South Plainfield, NJ) or A-M Systems glass 8250 (A-M Systems, Sequim, WA) using a Sutter P97 puller (Sutter Instrument, Novato, CA). The resistance of the patch pipette was 1.8-2.5 MΩ when filled with the standard internal solution. The tips of the patch pipettes were wrapped with Parafilm to reduce pipette capacitance. In whole-cell mode the capacity current was reduced by using the amplifier circuitry. Series resistance was 3.2 ± 0.8 MΩ (n = 122) when the patch pipette was filled with Cs-based internal solution for voltage clamp experiments, and 5.0 ± 1.4 MΩ (n = 27) when the patch pipette was filled with K-based internal solution for current clamp experiments. To reduce voltage errors, 70-80% of series resistance compensation was applied. In voltage clamp experiments a Cs-based internal solution was used to block outward currents through potassium channels. This solution contained (in mM): 125 CsCl, 10 NaCl, 2 MgCl_2_, 10 EGTA, 10 HEPES, 14 Tris-creatine PO_4_, 4 Mg-ATP, and 0.3 Na-GTP, pH = 7.2 with CsOH. In the experiments in Figure 5, 20 mM CsCl were replaced with 20 mM Tetraethylammonium-Cl to block outward current at depolarized potentials. In current clamp experiments a K-based internal solution was used. This solution contained (in mM): 135 K-methanesulfonate, 10 NaCl, 2 MgCl_2_, 0.1 EGTA, 10 HEPES, 14 Tris-creatine PO_4_, 4 Mg-ATP, and 0.3 Na-GTP, pH = 7.2 with KOH. Seals were formed in Tyrode’s solution containing (in mM): 151 NaCl, 2.5 KCl, 2 CaCl_2_, 10 HEPES, 13 glucose, pH = 7.4 with NaOH. After the whole-cell configuration had been established, cells were lifted off in front of an array of gravity-fed quartz flow pipes (I.D. 320 μm) that allowed rapid (<1 sec) exchange of external solutions. To isolate the TTX-R sodium current, the Tyrode’s solution was supplemented with 300 nM TTX and 30 μM CdCl_2_ in order to block the TTX-S sodium current and calcium current, respectively. The concentration of TTX used (300 nM) is sufficient to fully block the TTX-S sodium current while sparing the TTX-R sodium current, which requires about 40 μM for half-block [3,4,90]. Similarly, the amount of Cd^2+^ used (30 μM) to block the calcium current should have only minimal effect on the TTX-R sodium current [3,91,92].

Expression of TRPV1 channels in small DRG neurons was tested at the end of each experiment by challenging the neuron with 1 μM capsaicin and neurons were classified as TRPV1 positive if the inward current activated by capsaicin at – 80 mV reached at least −500 pA.

### Data acquisition and analysis

Currents and voltages were controlled and sampled using a Digidata 1440 A interface and pClamp 10 software (Molecular Devices, Sunnyvale, CA). Current or voltage signals were filtered at 10 kHz (−3 dB, 4-pole Bessel) and digitized at 50 kHz. Analysis was performed using pClamp 10 and Igor Pro (version 6, Wavemetrics, Lake Oswego, OR), using DataAccess (Bruxton, Seattle, WA) to import pClamp files in Igor. Small DRG neurons (diameter 18 to 25 μm) were initially selected by measuring the diameter from images captured to computer by a CCD camera Oly-150 (Olympus Imaging America Inc., Center Valley, PA) using a video acquisition card (dP dPict Imaging, Inc., Indianapolis, IN). A more accurate measurement of cell diameter was obtained from measurements of whole-cell capacitance assuming a membrane capacitance of 1 μF/cm^2^ and spherical shape. Cell capacitance was measured by integrating the average of 5–10 current responses to a – 5 mV step from −80 mV filtered at 10 KHz and acquired at 50 KHz. In Figure 5, conductance *(G)* was measured as *G = I/(V-V*_*res*_*)*, where *I* is the peak current, *V* is the voltage, and *Vres* is the reversal potential for sodium current. *V*_*res*_ (67.4 ± 2.7 mV, n = 13) was measured with series of steps from −100 to + 100 mV, increments of 10 mV. *G* is plotted normalized to *G*_*max*_, the peak conductance. The lines are best fits to the Boltzmann function: *1/(1 + exp[−(V-V*_*1/2*_*)/k]),* where *V* is the step membrane potential in millivolts, *V*_*1/2*_ is the half-maximal voltage in millivolts, and *k* is the slope factor in millivolts. Sodium channels availability was determined by using 500 msec prepulses from −120 to + 30 mV followed by a test pulse to −10 mV. The test pulse current is normalized to its maximal value. Solid lines are best fits to the Boltzmann function: *1/(1 + exp[(V-V*_*1/2*_*)/k])*, where *V* is the prepulse membrane potential, *V*_*1/2*_ is the half-maximal voltage, and *k* is the slope factor in millivolts. In current clamp experiments, reported voltages were corrected for the – 8 mV junction potential between the potassium methanesulfonate-based internal solution and the Tyrode’s solution present when zeroing pipette current. The junction potential was measured using a flowing 3 M KCl bridge [93]. Action potentials were elicited by injecting 0.8 sec ramps of current to 1 nA [56]. Usually action potential firing began within 150–200 msec. The peak of the first action potential was quite positive (average + 38 mV), but gradually decreased during current injection until only oscillations were observed. Only action potentials that had a peak ≥ 0 mV and amplitude (from peak to trough) of ≥ 30 mV were included in the analysis. All experiments were carried out at room temperature (22 ± 2°C). Data are reported as Mean ± SD. Statistical differences between data sets were analyzed using the t-test or one-way ANOVA, followed by post-hoc Dunnett analysis (for the data sets in Figure 3B). Differences were considered significant at *p < 0.05 or **p < 0.01.

## Abbreviations

DRG: Dorsal root ganglia; TTX: Tetrodotoxin; TTX-R: Tetrodotoxin-resistant; TTX-S: Tetrodotoxin-sensitive; TH: Tyrosine hydroxylase; EEDQ: Ethoxycarbonyl-2-ethoxy-1,2-dihydroquinoline.

## Competing interests

The authors declare that they have no competing interests.

## Authors’ contributions

WG performed research and analyzed data. ES analyzed data. MR performed research and analyzed data. MP designed research, performed research, analyzed data, and wrote the paper. All authors read and approved the final manuscript.
